# FarmDain, a Decision Support System for Dairy Sheep and Goat Production

**DOI:** 10.3390/ani13091495

**Published:** 2023-04-27

**Authors:** Malamati Louta, Panagiotis Karagiannis, Vasiliki Papanikolopoulou, Sotiria Vouraki, Evangelos Tsipis, Stergios Priskas, Georgia Koutouzidou, Alexandros Theodoridis, Socratis Dimitriou, Georgios Arsenos

**Affiliations:** 1 Telecommunication Networks and Advanced Services Laboratory, Department of Electrical and Computer Engineering, Faculty of Engineering, University of Western Macedonia, 50100 Kozani, Greece; 2 Laboratory of Animal Husbandry, School of Veterinary Medicine, Faculty of Health Sciences, Aristotle University, 54124 Thessaloniki, Greece; 3 Department of Agriculture, School of Agricultural Sciences, University of Western Macedonia, 53100 Florina, Greece; 4 Laboratory of Livestock Production Economics, School of Veterinary Medicine, Faculty of Health Sciences, Aristotle University, 54124 Thessaloniki, Greece; 5 Achilleas Socratis P.C., 1st Km. Amyntaio-Florina Rd., 53200 Amyntaio, Greece

**Keywords:** sheep, goats, decision-support tool, management, dairy industry, animal food value chains, animal health and welfare

## Abstract

**Simple Summary:**

Nowadays, there is a tendency for intensification in the sheep and goat sector to achieve higher production levels while improving farms’ profitability. Towards this end, the adoption of advanced technology is considered imperative. In this regard, we developed FarmDain, a novel web-based application for sheep and goat farming to help decision-making towards efficient farm management and planning in the dairy production value chain. In this study, FarmDain was used by a Dairy Factory which collaborates with over 100 dairy sheep and goat farms. In the presented case study, based on the reports provided by the app, feeding, culling and genetic selection decisions were made, improving overall farms’ performance and profitability.

**Abstract:**

Managing a milk zone in the dairy industry is demanding. Data necessary for efficient management are difficult to acquire because they usually must be collected in organized and standardized ways. On the other hand, software practices constantly provide new tools that can go beyond simple record-keeping practices and add value to the data. In this work, FarmDain is a novel web-based application for sheep and goat management. It aims to improve milk production and processing by digitizing the value chain in data acquisition, processing and visualization between dairy production businesses and their milk suppliers. FarmDain uses state-of-the-art software technologies to model the data collection process and provides a straightforward user interface to facilitate data processing and visualization. Using the app in a case study carried out for 12 months in a dairy sheep farm resulted in lower feeding cost per milked ewe by 5.5% when ewes were allocated into high and low milk production groups compared to the scenario of remaining in one single group. Furthermore, based on reports provided by the app, culling and genetic selection decisions were made to improve the overall farm performance. Similar practices were applied in all farms optimizing their productivity, which led to increased profitability for farms and the Dairy Factory.

## 1. Introduction

The dairy production value chain comprises highly complex consequent processes [1]. The current value and supply chains require high quantity and quality standards, usually under periodic review and update. Meeting these standards puts substantial pressure on dairy farmers and their labor force [2]. Furthermore, livestock production and management must deal with composite regulation issues and resource-demanding day-to-day procedures, which entail extensive record-keeping and decision-making [3] under uncertainty. Additionally, suboptimal productivity due to health and welfare issues, poor management practices and lack of genetic improvement are significant opponents of sustainable farming [4]. The latter problems are exemplified in the case of the dairy sheep and goat sector, especially in semi-intensive/extensive farming systems, characterized by a need for more professionalization and management training [5].

Decision Support Systems (DSS) [6] could help farmers to manage their enterprises more efficiently, achieving higher production and profitability [7]. This could benefit the sector’s overall sustainability, providing significant benefits to the dairy industry [8]. Most available DSS is focused on dairy cow farm management [9,10,11,12,13,14,15,16]. Although such systems could be potentially modified to include parameters tailored to the needs of small ruminant farming, none of the current solutions supports such capabilities. The dairy sheep and goat sector differs regarding animal needs and management practices applied (nutritional management, reproduction, farming systems). Hence, different methods and models would have to be incorporated into already available DSS for the dairy cow sector.

Regarding small ruminant farming, some DSS solutions have been developed to help farmers with specific issues such as grazing management [17], biodiversity, water, greenhouse gas emissions [18], and animal diseases [19]. Other available tools provide sustainability/economic performance assessments at the farm level [6,20,21,22] or facilitate individual-animal record keeping [23,24,25,26]. However, there is a notable shortage of tools with decision-support capabilities at the farm and animal levels. At the same time, farmers are the targeted users in all cases. Farmers’ low educational status and the lack of user-friendliness that characterizes most of the available DSS solutions hinder their adoption [27,28,29]. In this regard, DSS tools tailored to the needs of the dairy sheep and goat sector that enable data collection for important farm and animal parameters and support decision-making in the dairy value chain are warranted.

In this work, we introduce FarmDain, a novel web-based application developed to facilitate standardized data collection at the farm and animal level and to incorporate decision support tools and methods in the dairy value chain. FarmDain incorporates a role-based user hierarchy model and can be used by individual small ruminant farms or, more importantly, by any entity that manages several farms, such as the dairy industry. The latter allows for higher efficiency and accuracy in data collection and interpretation of results while also returning crucial information to the farmer for improving current practices. Such an approach could help increase production in small ruminant farms and benefit the overall dairy value chain. The above example is the scheme of the case study implemented to evaluate the application’s impact on sustainable dairy sheep and goat production. The results showed that FarmDain contributed to the process of informed decision-making, timely problem-solving, and efficiency in dealing with the complex operations and planning required in the dairy production value chain.

This paper is organized as follows. Section 2 describes the materials and methods used to design, implement and test FarmDain. Section 3 introduces the results achieved by detailing the features and functionalities of the application and how they were used to evaluate FarmDain through the use cases presented. Section 4 discusses how the results. Finally, Section 5 presents our conclusions.

## 2. Materials and Methods

### 2.1. System Architecture

**Figure 1:**
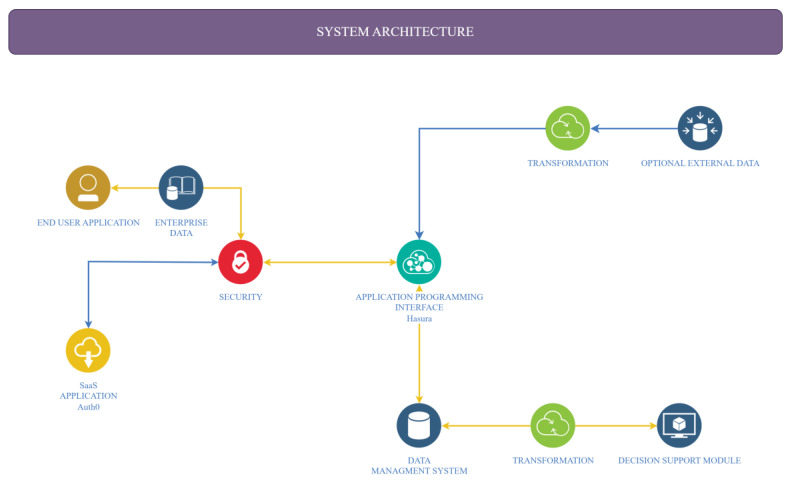
FarmDain’s Architecture.

FarmDain is a highly modular system comprising several independent components and services (Figure 1): the Data Management Service, the Application Programming Interface (API), the Authentication and Authorization Service, the Decision Support Module, and the End-User Application.

Although decision-making is a process that every manager utilizes, there is a lack of structured tools in the context of Greek farming. It is noted that a lot of bookkeeping in Greek farms is done either with analog means or with simple spreadsheets. The web application aims to provide a streamlined, localized, and easy-to-use tool that is available anywhere/anytime and can support farm administrators with everyday tasks and long-term farm management. The web application aims to be the starting point of a software platform that incorporates standardized data management and decision procedures. The DSS process is supported by normalized inputs, predefined data transformations and common outputs for multiple farms through a unified environment. The users have access, through the application, to analytics and visualizations, enabling them to make data-driven decisions more quickly and accurately.

The system architecture (Figure 1) presents the software tools and platforms that are integrated into the tool. Through the End-User Application, the user provides the enterprise data (e.g., data about animal characteristics, lactation data, and applied reproduction practices). Using Application Programming Interface (API), these data are stored in the Data Management System. Data can be utilized by the Decision Support Module in their raw form or after data transformations such as filtering, aggregation, and summarization. In addition, optional External data can be made available to the Data Management System after required transformations, such as data standardization through the API. All operations of the API are secured by the security module that utilizes the Auth0 service as a service.

As required in all management systems [30], FarmDain is easily accessible through its web and mobile user interfaces. The application enforces security through the Hypertext Transfer Protocol Secure (HTTPS) and encryption using the Transport Layer Security (TLS) protocol [31]. Its modular nature follows the microservice architectural pattern. Therefore, its components are distributed and loosely coupled. The microservice framework allows the integration of new tools and functionalities without requiring significant modifications. This makes the application flexible regarding extensibility and allows further expansion by adding and connecting new components as independent services.

FarmDain follows the cloud-first approach, meaning that all its modules are easily hosted in cloud environments which are secure, cost-efficient, and reliable in terms of either software or hardware failures. To this end, FarmDain uses containers, a virtualization technology that provides lightweight, standalone, executable software components.

This attribute leads to reduced costs and minimal downtimes. Furthermore, even if a component fails, whether software or hardware, the event rarely affects the entire system. On the contrary, such issues are usually confined to the module they originated from, while the rest of the application’s components remain operational.

### 2.2. Data Management Service

The Data Management Service is the backbone of the system. It facilitates data storage, querying, data entry, retrieval, updates, and Role-Based Access Control (RBAC). In addition, it is built to deploy its container. Therefore, it is highly scalable and, like all FarmDain’s components, can be used in container orchestrator environments which automate the deployment, management, scaling, and networking.

In this case study, we used PostgreSQL [32], an advanced Open-Source Relational Database Management System. This choice was tailored to the specific needs addressed by the case study, especially in building relationships between the collected data and optimizing the links between different data sources. Nevertheless, FarmDain’s architecture also supports the integration of various data sources such as NoSQL databases (e.g., MongoDB) or cloud-native ones (e.g., YugabyteDB, CockroachDB, etc.).

### 2.3. Application Programming Interface

The connection between the data layer and the End-User Application is achieved through Application Programming Interfaces (APIs). The APIs provide standardized functions to access the stored data and allow the application to interact with external software components and microservices.

A standard API is usually based on the representational state transfer (REST) [33] architecture style. The RESTful API is a well-proven way of communication between software systems that provides a uniform interface for transferring data in a standard format from the server to the client. However, as with every technology, REST also comes with drawbacks or constraints that must be considered before implementation (e.g., multiplexing multiple requests over a single TCP connection, having different resource requests for each resource file, server request uploads, long HTTP request headers that cause delays in webpage loading, lack of state, data over fetching/under fetching and absence of imposed security protocols, etc.). Hence, in FarmDain, we considered an alternative approach using GraphQL [34], developed internally by Facebook in 2012 and released publicly in 2015 [35]. GraphQL is a query language that uses the database schema to construct a complete description of the data stored in the data layer.

**Figure 2:**
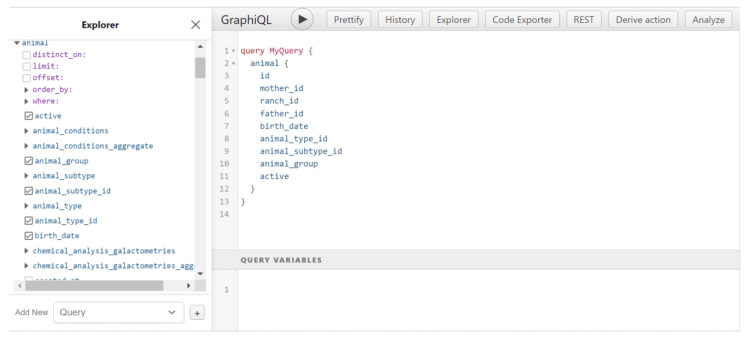
Hasura Interface for API testing.

During the implementation phase, as well as for deployment and testing, we used the Hasura GraphQL Engine [36] (Figure 2).

Therefore, FarmDain also adheres to the latest full-stack application standards by following the 3-factor app architecture pattern [37], which goes beyond a standards microservices-oriented solution, providing low-latency real-time queries, reliable event system and asynchronous, serverless functions. This approach forms the basis of a reliable, modern, and salable system where business logic is handled through events. Additionally, our approach facilitates asynchronous serverless operations that allow for minimal DevOps and low-cost and effective scaling [38,39]. Thus, FarmDain can incorporate multiple remote schemas that allow the use of data from various sources. It can also provide event triggers on database tables that allow interaction when an event occurs, allowing for more sophisticated data pipelines when certain events occur, such as new data insertions or data updates.

### 2.4. Authentication and Authorization Service

Authentication and Authorization are interrelated concepts and form the core of the security aspects of a software application [40]. However, developing secure, tested, and maintainable authentication systems is very difficult because security protocols are very difficult to implement, update and maintain by small teams. In addition, web-based systems are constantly threatened by cyber-attacks that can harm their users and lead to data theft and financial harm. To leverage these barriers, FarmDain uses a very popular cloud Authentication Service nuth0 [41]. Auth0 is an authentication and authorization solution that provides an adaptable framework for complex security requirements through the Software as a Service (SaaS) licensing and delivery model.

Furthermore, the authentication and authorization process are based on using JSON Web Tokens (JWT), an open standard [42] that is compact and secure. The information contained within the JSON object is digitally signed by a unique secret key using HMAC or RSA algorithms, and thus it can be verified and trusted.

JWTs are used for both Authentication and Authorization [43] by utilizing the ID and Access tokens, respectively. Both are small data structures that can provide information about the user (ID token) and the privileges assigned to the user by the administrator. The access token is then used to give access to different resources.

**Figure 3:**
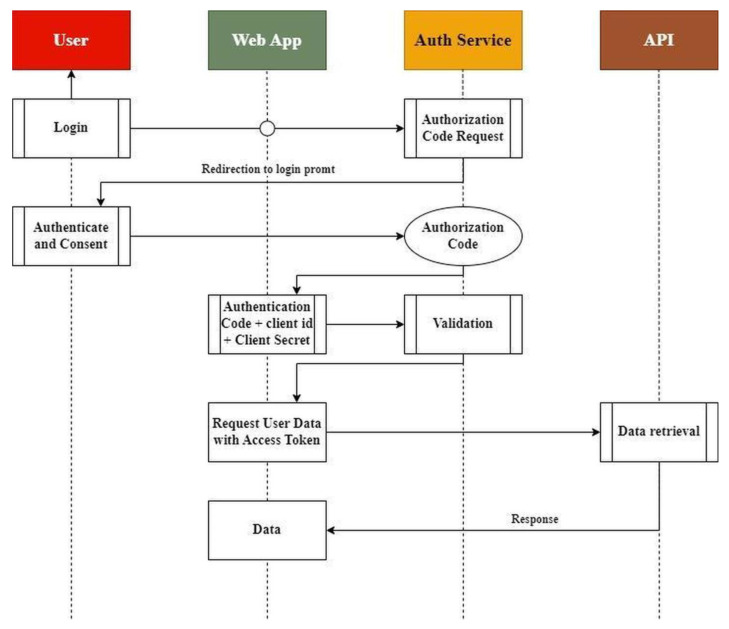
Authentication and Authorization flow.

The Authentication and Authorization flow, presented in Figure 3, allows a user who requires a login to the End-User application of the platform to exchange their credentials for an authorization code that can be validated back to the service. After validation, an access token is returned to the application by the service, which can be used in every data request. Thus, every request is secured and contains specific info about the caller. This allows the API to respond with the appropriate data after internal business logic has cross-checked the predefined data permissions.

### 2.5. Decision Support Tools

Data-driven DSS systems [44] facilitate the analysis of large amounts of structured data. In FarmDain, this functionality is built within the Data Management Service and the End-User Application. It is accessible to the end-user through distinct views of data on the front-end application. The main feature of the DSS system delivered through the application layer is to visualize flows and information derived from transformations applied to the recorded data.

### 2.6. End-User Application

FarmDain was developed as a web application. Its End-User Interface is the entry point for data collection, management, and representation. Therefore, the application has zero installation requirements since it is deployed through cloud servers and delivered by all modern web browsers. Additionally, a web application has reduced maintenance costs because every update happens only in the context of the server and can provide cross-platform availability.

**Figure 4:**
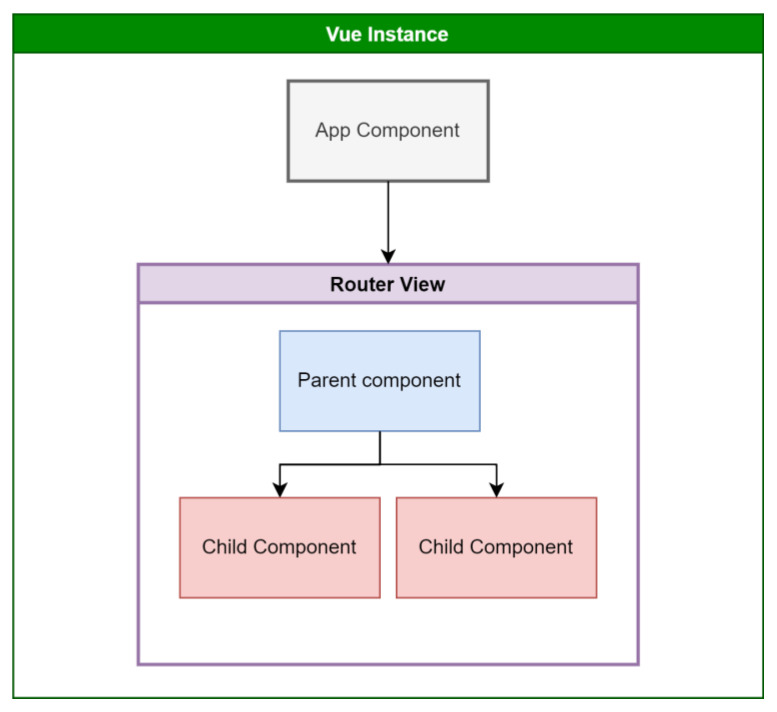
Vue.js Components.

Development was done with Vue.js, a JavaScript Framework [45], on top of JavaScript, HTML, and CSS tools. Vue.js is a performant, approachable, versatile framework for building web user interfaces. This approach automates the development and reduces the overhead of common activities during the software’s lifecycle. It also uses reusable components (Figure 4), which are reusable blocks of code acting as building blocks for an extensive application.

The Vue instance has a base App component mounted onto a DOM element and organized into a tree of nested, reusable components. Thus, the End-User Application follows the Model–View–ViewModel architecture [46], where the Vue instance component acts as the ViewModel, handling the communication between the part responsible for the representation of the data called View and the part that is still responsible for accessing different data sources called Model [47].

FarmDain uses built-in page routing, the basic functionality that allows for a responsive and crisp user experience [48]. The user is directed to different pages without reloading the whole page. This gives the feeling of a desktop application and reduces load times. In addition, FarmDain handles state management, either on a component level or on an application level. External libraries are not needed since the functionality is also built-in. Finally, caching minimizes server-client calls making the application faster than a traditional web application.

The End-User Application can be delivered either as a Single Page App (SPA), a type of application that uses only one HTML web page as a shell for all the application’s web pages [49] or a Progressive Web Application (PWA) [50]. This gives the advantage of reducing development to a single code base. In addition, a native-like application (e.g., Android Application) can also be developed, if required, with reduced costs.

FarmDain can also be used from Android systems through any mobile browser (chrome, firefox, edge etc.). Nevertheless, the approach to developing FarmDain requires only front-end development for the mobile app since the backend (data layer and API) is already present. Furthermore, the FarmDain application is developed as a thin client web app. This is a type of web application that relies heavily on server-side processing and minimal client-side resources. Since the application runs entirely in a web browser, it can be accessed from any device with a modern web browser, regardless of the operating system. Moreover, developing a native application first would require choosing one of the two major vendors (android/iOS) or adding to the development and maintenance costs by sustaining multiple code bases, one for each platform (Lines).

### 2.7. Collected Data

Data collection is a relatively simple process. First, FarmDain gathers and groups farm data pertaining to sheep and goat farming, such as production, reproduction strategies, feeding, grazing, animal health and welfare status, and laboratory analyses on the farm and animal level (All variables are available in Dataset S1). Regarding the latter, FarmDain extensively uses the animal ear tag as the unique identifier tagging system, which is already legally required by livestock farms, as the core of its record-keeping protocols [9]. Furthermore, data is gathered manually whenever management decisions need to be made, either on the farm or animal level. Thus, the collected data can provide vital insights into the milk value chain, as they contain various levels of information. In detail, the collected data consists of the following:

Data on Farm Level

- General farm information: (Farm ID, location, farming system).
- Labor data (number of workers, family labor used, full-time/part-time-seasonal labor, hours of work per month).
- Livestock data (breed type, flock size, replacement rate, total number of newborns, total number of animals slaughtered or sold, number of deaths).
- Milk production traits and quality of produced milk (total milk production, average milk yield per animal, lactation period length, milk composition (fat, protein, lactose, and solids-non-fat (SNF)) and microbiological parameters including Total Viable Count (TVC) and Somatic Cell Count (SCC)).
- Grazing and feeding practices (feedstuffs used and quantities in all production stages, chemical composition).
- Reproduction management practices and techniques [onset of the breeding season, the onset of lambing/kidding period, prolificacy, number of abortions, estrus synchronization, artificial insemination (AI).
- Suckling period management (natural or artificial rearing).
- Milking (hand or machine milking, number of milkers, milking per day, milking duration, use of gloves, post-dipping, cleaning and disinfection, and other practices).
- Stable (type of housing and size).
- Veterinary and general practices (electronic identification systems, claw trimming, intramammary dry-treatment, anthelmintic treatment, vaccination program, genotyping for resistance to scrapie and other diseases).
- Economic data for income and variable costs (subsidies, prices of milk, meat, feeding and animal sales, costs of labor, utility, transportation, milking parlor, land, veterinary and other).
- Data on the Animal level
- Animal (species, genealogy, age, lactation number).
- Welfare and health indicators (head: skin lesions, injuries or abscesses, limbs: overgrown claws and arthritis, udder: asymmetry, skin lesions, abscesses, and fibrosis, records of California Mastitis Test (CMT)).
- Reproduction strategies (selected breeding method, dates of (i) natural mating, (ii) AI, (iii) entrance of males after AI, (iv) insertion of progestogen sponges, (v) rams’ ID used in AI);
- Lambing/Kidding period (number and gender of newborns per animal, date of weaning);
- Milk recordings (date of milk recording, daily milk yield and corresponding milk fat-, protein-, lactose-, and SNF content).
- Ultrasound data (pregnancy, pathological findings).
- Data at this detailed level can provide valuable information about each animal and the milk produced. This type of information is analyzed by the DSS module to provide insights about the enterprise operation and allow for milk traceability on a very detailed level.

Independently of the hierarchical level of data acquisition, all gathered information is stored as database entities (tables) with predefined relationships of appropriate cardinalities. Data are available to the End-User application of the Platform but are also available to third-party applications that, given the appropriate permissions, can further utilize them.

Data is uploaded through the web application by the end-user. The application can read Microsoft Excel spreadsheets provided by the user using a standard file-select browser interface. The next step transforms the data into JSON Objects in the client and transfers them to the data management service. The Application Programming Interface enables this communication between the front-end application and the Data management service utilizing HTTP calls. It provides back responses of success or failure of the transmission procedure.

### 2.8. A Case Study Application

For the demonstration of the FarmDain application, a case study from a dairy sheep farm collaborating with the dairy industry is presented. Our aim was to increase the annual milk production of dairy ewes through an effective nutritional plan and reproductive management practices, improving the profitability of the farm and the Dairy Factory. This farm comprised 174 milked ewes of the Lacaune breed reared under intensive conditions. A veterinarian cooperating with the dairy industry visited the farm to collect data regarding general farm and animal-level management, as asserted earlier in Section 2.7 (questions with personal or sensitive data were not included). Furthermore, individual milk recording was performed monthly (*n* = 5, every 30–45 days) during the milking period after lamb weaning. Collected data were inserted manually into the FarmDain application and assessed accordingly (Dataset S2). Following each milk recording and based on the average total milk production reports provided by the FarmDain application, ewes were allocated into a high or a low milk production group. Both groups were fed with alfalfa hay, maize silage and concentrate feed according to INRA recommendations to meet nutritional requirements. Feeding costs per liter of produced milk were calculated for two scenarios; when ewes were allocated to high or low groups compared to the case ewes had remained in a single group. In the latter case, feeding is based on average milk production, and the ration provided does not meet nutrient requirements for protein and energy, especially in high-producing animals, resulting in lower milk yields. Furthermore, according to reports and statistics from the FarmDain application, ewes with the highest milk yields were selected for AI.

## 3. Results

### 3.1. User Interface

**Figure 5:**
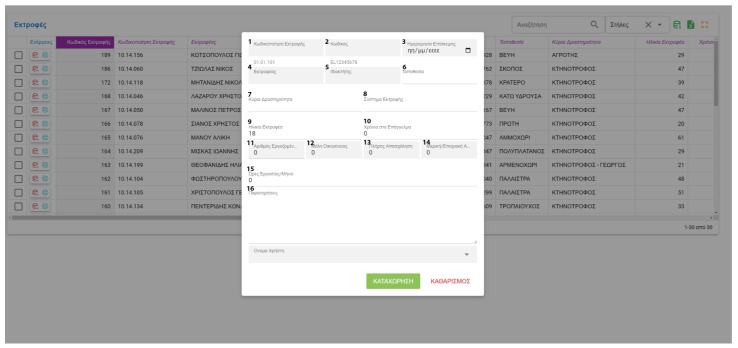
New farm input data (^1^ Internal Farm Code, ^2^ Farm Code, ^3^ Visit Date, ^4^ Farmer, ^5^ Farm Owner, ^6^ Farm Location, ^7^ Main Activity, ^8^ Farming System, ^9^ Farmers’ Age, ^10^ Farmers’ Experience Years, ^11^ Number of Employees, ^12^ Family Members employed, ^13^ Full-Time Employees, ^14^ Part-time employees, ^15^ Average Work Hours/month, ^16^ Notes).

**Figure 6:**
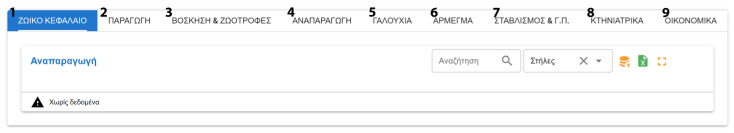
Farm level data tables (^1^ Livestock, ^2^ Labor, ^3^ Milk Production, ^4^ Grazing and Feeding, ^5^ Reproduction, ^6^ Milking, ^7^ Stabling and General Practices, ^8^ Veterinary Information, ^9^ Economic Parameters).

**Figure 7:**
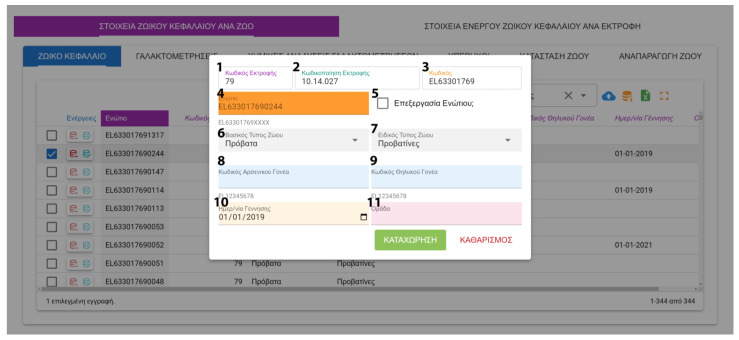
Animal level data management *(*^1^ Internal Farm Code, ^2^ Identifier Code, ^3^ Farm Code, ^4^ Animal Tag, ^5^ Edit Tag, ^6^ Animal Species, ^7^ Animal Category, ^8^ Male Parent Tag, ^9^ Female Parent Tag, ^10^ Birth Date, ^11^ Animal Group).

**Figure 8:**
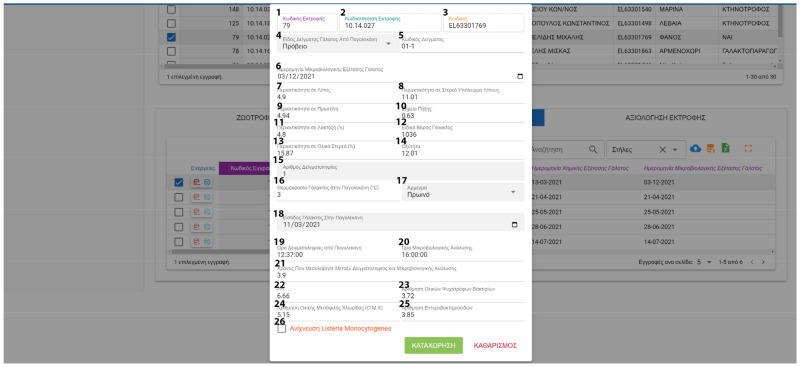
Milk Analysis Data (^1^ Internal Farm Code, ^2^ Identifier codes, ^3^ Farm Codes, ^4^ Sample Type, ^5^ Sample Code, ^6^ Sampling Dates, ^7^ Milk Fat Content (%), ^8^ Milk Solid Non-Fat Content (%), ^9^ Milk Protein Content (%), ^10^ Freezing Point Depression (FPD), ^11^ Milk Lactose Content (%), ^12^ Milk Density, ^13^ Milk Total Solid, ^14^ Milk Acidity, ^15^ Sampling number, ^16^ Temperature in Milk Tank, ^17^ Milking types, ^18^ Date of Milk Insertion in Milk Tank, ^19^ Sampling Time rom Milk Tank, ^20^ Time of Microbiological Analysis, ^21^ Time between Sampling And Microbiological Analysis, ^22^ Milk pH, ^23^ Psychrotrophic Bacteria Concentration, ^24^ Total Plate Count, ^25^ Enterobacteriaceae Concentration, ^26^ Listeria Monocytogenes).

FarmDain has a user-friendly interface for the enterprise manager, which includes the following:

1. Login. Initially, the users must access the application with their unique credentials.
2. Farm management. New farms can be created, or already recorded farms can be edited (Figure 5). Data collected are used to identify and describe each farm.
3. Management of farm-level data. These kinds of data input are made available to the user after the initial farm input and provide detailed information about livestock, labor, milk production, grazing and feeding, reproduction, milking, stabling and general practices applied in the farm, veterinary information, and economic parameters. Data input and editing are achieved with a unified interface and easily identified buttons (Figure 6).
4. Management of animal data; Following the same interface, the user can provide or edit information on the animal level Information includes general livestock info, reproduction data, milk recordings, and laboratory and ultrasound results (Figure 7). Milking recordings and ultrasound results can also be viewed per ranch on a different tab for easy access.
5. Management of laboratory analysis data; The user can input or edit results about the chemical composition of feedstuffs and milk and the microbiological profile of milk and dairy products (Figure 8).

### 3.2. Main Features

#### 3.2.1. Reports

**Figure 9:**
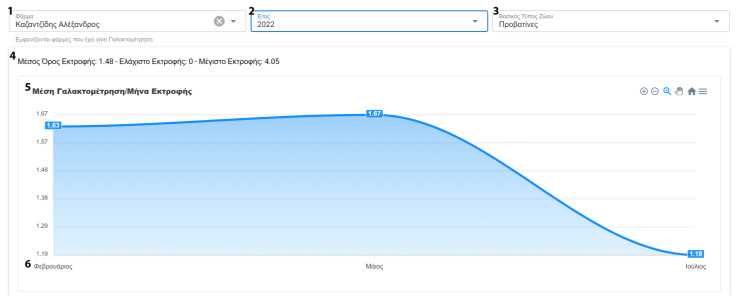
Report average milk production per year/month (^1^ Farm Selection, ^2^ Year Selection, ^3^ Animal Type Selection, ^4^ Total and Average Milk Yield for Selected Farm, ^5^ Average Farm Milk Yield per month, ^6^ Month).

**Figure 10:**
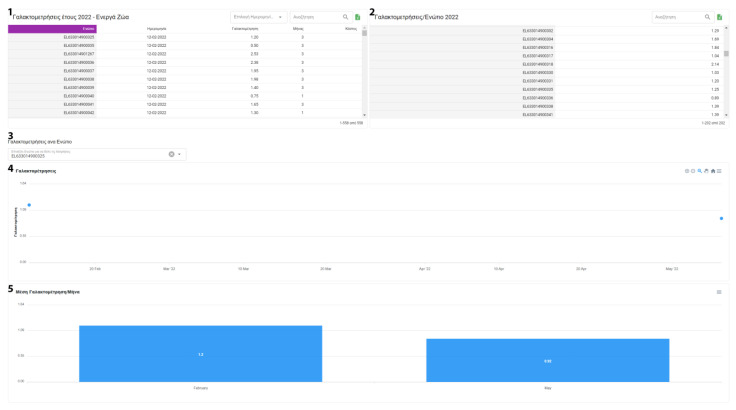
Report of average, total, and daily milk production per animal (^1^ Milk Yield per animal by year, ^2^ Average Milk Yield per Animal by year, ^3^ Animal Selections, ^4^ Graphical Representations of Milk Yield per animal, ^5^ Graphical Representations of Average Milk Yield per Animal by month).

After data collection, the user has access to numerous statistics. These statistics are present in various forms, such as graphs and tables. When applicable, this information can be filtered and grouped by farm or animal categories. The system’s reports contain information about milk recordings and general farm management indicators. Milk statistics include information about the average milk yield per farm. Moreover, individual data on total, average and daily milk yield are also calculated (Figure 9 and Figure 10).

**Figure 11:**
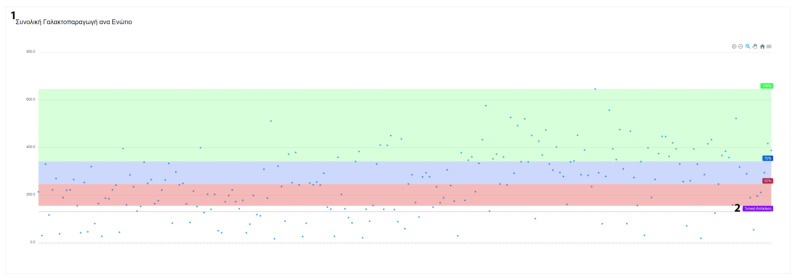
Report of total milk production for each animal by lactation period *(*^1^ Total Milk Yield per Animal, ^2^ Standard Deviation).

Additionally, FarmDain can provide reports on udder health issues and total milk production for each animal by lactation period (Figure 11). To provide the user with a better overview of each animal, the information depicted is further enhanced with the dates that diseases occurred during each animal’s recorded period. In the livestock reporting section, the user has an up-to-date view of the active and inactive (e.g., deceased) livestock population and the total recorded livestock population.

#### 3.2.2. Traceability

**Figure 12:**
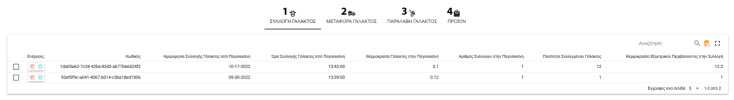
Milk Traceability (^1^ Milk Collection, ^2^ Milk Transportation, ^3^ Milk Reception, ^4^ Dairy products).

The user is asked to input data about the milk flow to the enterprise. These include information about the milk collection at the farm, transport, and reception at the enterprise. Using the same standardized interface, information about collection times, storing, transfer and pickup temperatures, milk composition and presence of antibiotics is recorded using a stepped interface flow (Figure 12).

**Figure 13:**
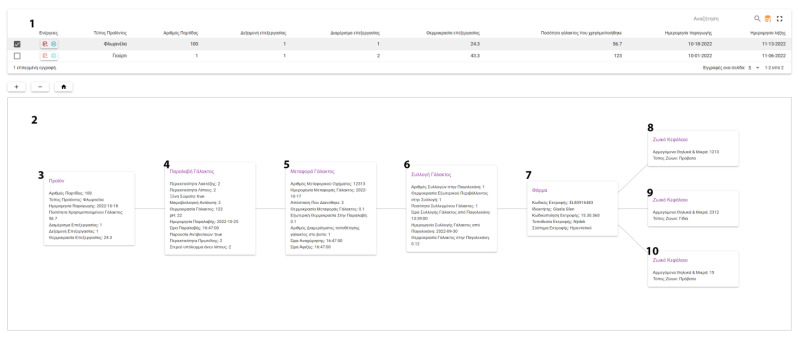
Traceability (^1^ General Data, ^2^ Product Lot Tracing Tree, ^3^ Dairy products, ^4^ Milk Reception, ^5^ Milk Transportation, ^6^ Milk Collection, ^7^ Farm codes, ^8,9,10^ Livestock data).

In the final stage, each product lot relates to the picked-up milk and a complete path in the form of an expandable tree is presented to the user (Figure 13).

### 3.3. Case Study Results

Feeding cost per liter of produced milk was reduced by 5.5% when ewes were allocated into high and low milk production groups compared to remaining in one single group; the highest reduction was observed in the second and fourth (0.04 €/L) milk recording. Moreover, 12 and 16 ewes in the flock were identified and culled early due to low productivity (<1 L/day) and udder health issues, respectively. Based on application reports, 50% of milked ewes with the highest milk yield were selected for AI to produce genetically superior lambs for replacement. As a result, the milk production of these ewes was higher by 56.1% (184 L) compared to the flock’s average production.

## 4. Discussion

As asserted in the introduction, several challenges threaten the sustainability of the dairy production sector. Considering these challenges, we designed and developed FarmDain, a web-based DSS that facilitates data collection at the farm and animal level in dairy sheep and goat farms. The aim is to help decision-making towards efficient farm management and planning in the dairy production value chain.

FarmDain DSS was developed within the project “Development of an integrated milk management system, using new technologies, for quality assurance and milk product traceability in the milk industry” (FarmDain). Specifically, the tool was designed to accommodate the needs of a dairy industry located in a less-favoured area of northwestern Greece. Previous research indicated that one of the main barriers to adopting new technologies and especially applications is the low educational status of farmers [29]. Therefore, contrary to other available tools, FarmDain incorporates a role-based user hierarchy model that enables the dairy industry to be the user of the application. This approach allows for the timely and accurate input of collected data in the application while ensuring that critical information is returned to each farmer to establish a more efficient management plan and make informed decisions for higher productivity and improved animal health and welfare status.

FarmDain considers all important farm parameters, allowing for a holistic management approach. It goes beyond other DSS tools focused on helping farmers deal with specific problems [17,18,19]. Moreover, FarmDain collects data at the farm and animal level, further differentiating it from other available solutions. Specifically, most DSS tools facilitate management decisions through sustainability assessments based on farm data [6,20,21,22]. Regarding individual animal recording, available tools are limited, and only in a few cases do they provide precision farming capabilities utilizing electronic identification systems [23,24,25]. FarmDain uses the electronic ear tag as the unique identifier of each animal. This enables accurate performance recording and monitoring of health and welfare issues, thus facilitating genetic selection practices and culling decisions [51,52].

Moreover, this is an efficient way of ensuring the traceability of animals and their products, which is essential for the dairy industry. Supporting traceability from farm to industry is another important feature of FarmDain that distinguishes it from other available DSS in the sector. Specifically, most systems regarding the traceability of dairy products focus on collecting data at the farm during transportation or processing [25,53,54].

Based on the features described above, several impacts of the FarmDain application can be identified. First, at the farm level, users are given an overview of all important farm management practices (flock size, production, feeding and grazing, reproduction, milking, veterinary practices, and economic figures). In this regard, the proposed system facilitates the evaluation of current farm management and the uptake of decisions for production optimization and profit maximization. At the animal level, management and genetic selection practices are facilitated through accurate pedigree recording and individual information on milk production (daily, average, and total), health, welfare, and reproduction efficiency. Specifically, based on such information, (i) the most productive animals can be identified, (ii) high and low milk production groups can be distinguished and different feeding practices applied, and (iii) culling decisions can be made according to milk production, reproductive performance, and health issues. Therefore, FarmDain supports genetic improvement in dairy sheep and goat farms. The latter has been identified as one of the most essential best practices for increasing farm profitability and the overall efficiency of the sector [55]. Finally, FarmDain allows for the traceability of dairy products considering information on the flow of milk from the farm to the industry, its processing and labeling. Traceability of dairy products is mandatory within the EU (Regulation (EC) No 178/2002) [56]. Hence, FarmDain helps the dairy industry adhere to relevant regulations and guarantees its products’ transparency, quality, and safety [54].

Such impacts were demonstrated in our case study application. For the case study, one farm was selected as representative of those collaborating with the dairy industry; similar management practices were implemented in the other farms following the use of FarmDain. Based on collected data and reports provided by the application, the farm could identify the high from the low-producing animals, which were grouped and fed separately according to their production level. This resulted in a decrease in feeding costs by 5.5%. Moreover, cervical AI was implemented in the highest-producing animals (50% of ewes) to increase genetic gain.

Additionally, animals with poor udder conformation were identified and marked as unsuitable for keeping progeny. Finally, animals with low milk production and/or health issues were placed and culled. Such results confirm that FarmDain can help farmers towards more efficient farm management and higher production.

FarmDain uses a role-based user model, providing improved accuracy in data collection and interpretation. Therefore, numerical, and behavioral uncertainty [57] are addressed through cross-data checks, reports, and data analysis after data entry. Additionally, during the testing phase, in which the application was under continuous scrutiny, we faced and resolved various errors in software development, operations and system component interaction. Moreover, the case study provided a solid testbed for identifying, categorizing, and addressing random (e.g., misinterpretations in metrics of quantity parameters) and ambiguity (e.g., human communication errors) uncertainties.

Currently, FarmDain operates in the Greek language. Therefore, one of the main priorities for the future is to translate the application into English to enable its potential use by other interested dairy industries in the EU. Moreover, the developing group will continue working towards the long-term utility of the application by monitoring the functionality of the system and maintaining the website page. Finally, to better meet the needs of the dairy sheep and goat value chain, the possibility of including new variables will be evaluated in collaboration with the dairy industry, the associated farmers, and their consultants. Special emphasis will be given to including environmental sustainability indicators that have been highlighted as imperative for adjustment of the livestock sector to new environmental policies as well as welfare indicators related to the animal environment, including microclimatic parameters [58].

## 5. Conclusions

FarmDain is a user-friendly, novel web-based application for sheep and goat farm management and planning in the dairy production value chain. Specifically, FarmDain enables data collection at the farm and animal level and provides decision support capabilities through powerful reports with graphs that help farms to optimize their overall performance with economic benefits. In our study, FarmDain was primarily used by the dairy industry to manage its collaborating farms efficiently. Data collection and the following assessment based on reports provided by the app led to identifying the main problems in each farm and making appropriate management decisions. Hence, the overall performance and profitability of both farms and the dairy industry significantly increased.

## Data Availability

Data presented in this study is contained within the article and Supporting Material.

## Supplementary Materials

The following supporting information can be downloaded at: https://www.mdpi.com/article/10.3390/ani13091495/s1, Dataset S1: Total dataset with variables included in the FarmDain decision support system; Dataset S2: Dataset of milk recordings used in the case study.
